# The Complex Trauma Questionnaire (ComplexTQ): development and preliminary psychometric properties of an instrument for measuring early relational trauma

**DOI:** 10.3389/fpsyg.2015.01323

**Published:** 2015-09-01

**Authors:** Carola Maggiora Vergano, Marco Lauriola, Anna M. Speranza

**Affiliations:** ^1^Department of Dynamic and Clinical Psychology, Sapienza UniversityRome, Italy; ^2^Department of Developmental Processes and Socialization, Sapienza UniversityRome, Italy

**Keywords:** Complex Trauma Questionnaire, relational trauma, attachment, assessment, psychometric properties

## Abstract

Research on the etiology of adult psychopathology and its relationship with childhood trauma has focused primarily on specific forms of maltreatment. This study developed an instrument for the assessment of childhood and adolescence trauma that would aid in identifying the role of co-occurring childhood stressors and chronic adverse conditions. The Complex Trauma Questionnaire (ComplexTQ), in both clinician and self-report versions, is a measure for the assessment of multi-type maltreatment: physical, psychological, and sexual abuse; physical and emotional neglect as well as other traumatic experiences, such rejection, role reversal, witnessing domestic violence, separations, and losses. The four-point Likert scale allows to specifically indicate with which caregiver the traumatic experience has occurred. A total of 229 participants, a sample of 79 nonclinical and that of 150 high-risk and clinical participants, were assessed with the ComplexTQ clinician version applied to Adult Attachment Interview (AAI) transcripts. Initial analyses indicate acceptable inter-rater reliability. A good fit to a 6-factor model regarding the experience with the mother and to a 5-factor model with the experience with the father was obtained; the internal consistency of factors derived was good. Convergent validity was provided with the AAI scales. ComplexTQ factors discriminated normative from high-risk and clinical samples. The findings suggest a promising, reliable, and valid measurement of early relational trauma that is reported; furthermore, it is easy to complete and is useful for both research and clinical practice.

## Introduction

Over the past two decades, evidence regarding the harmful impact of early maltreatment has accumulated significantly. Two-thirds of adolescents and adults report having suffered from child relational trauma, which represents a root cause of major public health issues (Copeland et al., 2007; van der Kolk and d'Andrea, 2010). Previous research has primarily focused on single types of maltreatment sequelae; however, most maltreated children experience more than one form of abuse and neglect (Kessler, 2000; Spinazzola et al., 2005; Pynoos et al., 2008). Indeed, in literature on child abuse, the focus has shifted from individual and specific conditions of risk to a multi-determined etiology of the traumatic experience: initially, the literature focused on a punctiform trauma but now investigates the concept of complex trauma, describing a traumatic developmental context in which the child is immersed. Complex trauma can be understood as experiences of cumulative, chronic, and prolonged traumatic events, most often of an interpersonal nature, involving primary caregiving system, and frequently arising in early childhood or adolescence (Cook et al., 2005; Courtois, 2008). Till date, growing evidence has acknowledged the co-occurrence of multiple types of severe adversities (Mullen et al., 1996; Higgins and McCabe, 2001; Diaz et al., 2002; Clemmons et al., 2003; Dong et al., 2004; Stevens et al., 2005; Arata et al., 2007; Finkelhor et al., 2007, 2009; Turner et al., 2010; Greeson et al., 2011; Trickett et al., 2011) and their greater risk for subsequent trauma exposure and cumulative clinical impairment compared with singly traumatized youth (Schumm et al., 2006; Finkelhor et al., 2007, 2009; Cloitre et al., 2009; Margolin et al., 2009; Shen, 2009; Heim et al., 2010). However, numerous studies highlight the additive effect of child and adolescent multi-type maltreatment on later symptom complexity and psychopathology, including internalizing (Danielson et al., 2005; Schumm et al., 2006; Anda et al., 2007; Sachs-Ericsson et al., 2007; Widom et al., 2007; Ford et al., 2010), externalizing (Brown and Anderson, 1991; Herrenkohl et al., 1997; Finkelhor et al., 2009; Ford et al., 2009, 2010; Shen, 2009), and trauma symptoms (Boney-McCoy and Finkelhor, 1996; Mulder et al., 1998; Schaaf and McCanne, 1998; Finkelhor et al., 2007, 2009; Vranceanu et al., 2007; Shen, 2009; Ford et al., 2010). Following this large amount of studies, it is understandable that trauma may be referred not only as a present-or-absent construct but also includes dimensional aspects, considering the multiplicity of maltreatment forms observed as well as the frequency of traumatic exposure.

Literature on attachment has generated stimulating outcomes regarding early traumatic experiences and how they shape later responses in adulthood, becoming a framework that enables a more sophisticated comprehension of the relational trauma sequelae on mental health (Lyons-Ruth and Jacobvitz, 2008). Occurrence of abuse and neglect in early life may interfere with the development of a healthy secure attachment relationship (Baer and Martinez, 2006). Research findings suggest that traumatized children are at risk for attachment disruption, specifically developing an insecure disorganized attachment pattern (Lyons-Ruth et al., 1987; Carlson et al., 1989; Cicchetti and Barnett, 1991; Beeghly and Cicchetti, 1994; Barnett et al., 1999; van IJzendoorn et al., 1999; Cicchetti et al., 2006; Cyr et al., 2010). Similarly, during the administration of the Adult Attachment Interview (AAI; George et al., 1984; Main et al., 2003), the surfacing of memories of attachment-related traumatic experiences is not rare. In such cases, disorganized attachment pattern is most typically detected in adult respondents with a history of complex trauma (Lyons-Ruth and Jacobvitz, 2008; Bakermans-Kranenburg and van IJzendoorn, 2009; Murphy et al., 2014).

The assessment of complex trauma is by definition “complex,” as it involves both a delineation of a wide range of traumatic events as well as their appraisal, and because these types of experiences rarely occur in isolation and are highly interrelated (Felitti et al., 1998; U.S. Department of Health and Human Services, Administration on Children, Youth, and Families, 2011). Ascertaining the presence and degree of early maltreatment required for clinically based studies and epidemiological research has been enabled by observer-rated interviews and self-report questionnaires, though retrospective reports usually provide underestimates of the trauma incidence (Hardt and Rutter, 2004). Self-reports generally inquire about limited types and generate quantitative rating of trauma, and are unable to detect when defense mechanisms may distort responses (Ravitz et al., 2010). However, this format requires less time to complete and may elicit more disclosure facilitating valid responses to questions on sensitive issues (Newman et al., 1996). Several guided or semi-structured interviews have been developed to measure a broader range of potential trauma areas to reduce response biases (Roy and Perry, 2004), allowing investigators to obtain uniform information in an interpersonal interaction context. Nevertheless, these instruments are usually time-consuming and require administration and scoring by trained investigators.

Given the pervasiveness of the trauma reported and the outcomes that arise, these findings emphasize the vitality of a comprehensive assessment of early trauma history for making appropriate service recommendations and interventions within child and adolescent welfare (Kisiel et al., 2009; Briggs et al., 2013). Despite several measures that have been created to assess the occurrence of childhood trauma, researchers often base assessment tools on a narrowed conceptualization of maltreatment and focus on creating a single or limited abuse types-instrument (Bremner et al., 2007; DiLillo et al., 2010), making it difficult to extend the investigation of the multiple aspects of traumatic experience and to compare the impact of specific forms of trauma. Moreover, most studies use a priori categorical or dimensional descriptions of complex trauma, while only few define polyvictimization empirically using statistical techniques. Lastly, although evidence regarding the nature and frequency of traumatic exposure is significant to research and practice on child maltreatment (Manly et al., 2001; English et al., 2005), many instruments restrict assessment to the occurrence of trauma in a dichotomous (presence vs. absence) classification. In response to these gaps in measurement, this study aimed to develop a retrospective questionnaire concerning early history of interpersonal trauma and to outline its preliminary psychometric characteristics by presenting data which support its reliability and validity. In this paper, the Complex Trauma Questionnaire (ComplexTQ) was particularly applied to AAI transcripts conceived as a stimulus which would enable activation of the attachment system at emotional levels through an intensive series of probes regarding the interviewee's history with the attachment figures (Dozier and Kobak, 1992; Dias et al., 2011; Farina et al., 2014).

## Materials and methods

### Development and features of the Complex Trauma Questionnaire

The Complex Trauma Questionnaire (ComplexTQ) was developed to enable researchers and clinicians to measure adverse childhood experiences displayed in their major forms and frequency of occurrence, to cover multidimensional aspects of complex trauma, including child neglect which represents one of the most widespread forms of maltreatment (Gilbert et al., 2009; U.S. Department of Health and Human Services, Administration on Children, Youth, and Families, 2011), which appears as a deceitful phenomenon and is less easily detectable and assessable compared with active types of abuse.

The ComplexTQ was designed in both clinician and self-report versions^1^. In this study, the questionnaire is completed by a clinician and is applied to interview transcripts for evaluation of trauma history prior to age 15. Item construction was based on clinical experience and performed following an extensive review of the literature on child abuse and neglect. Clinician responds to a series of specific screener questions that reveal whether the participant experienced a range of caregiver's behaviors encompassing acts of both commission and omission. To capture the intensity of these experiences, clinician provides ratings on a 4-point Likert scale (from 1 = never to 4 = often) reflecting how frequently the episodes occurred, except for the last item concerning the occurrence of significant bereavements (yes/no). Thus, scoring allows detecting presence and severity (sum and mean of frequency scores) of each maltreatment type. Cumulative occurrence across all single forms of traumatic experience is provided through a total score. Given research and theory suggesting that the caregiver gender may be relevant to trauma effects (Schore, 1999; Briere and Rickards, 2007), scores are differentiated between relationship with the mother, that with the father as well as with another significant figure.

Based on the literature on childhood trauma, nine domains of interpersonal maltreatment experiences were considered and operationalized. Items regarding the *Neglect* domain are intended to measure withdrawal of caretaking and failure to respond and engage in behavior that is necessary to meet the developmental needs of a child, such as physical needs (e.g., providing food, clothing, and medical treatment), supervision and educational needs (e.g., checking on friendship environment and leisure activities, monitoring school attendance, or assisting with homework), and emotional needs (e.g., demonstrating affection, support, and companionship). These conducts attempt to capture the heterogeneity of child neglect, ranging from a caregiver who is unavailable, inattentive, uninvolved, or psychologically inaccessible (e.g., lacking of love expressions) to the one whose acts involve abandonment of the child. Consequently, the child may feel unloved, unnoticed, or invisible to the caregiver. *Reject* items describe a cold or hostile caregiving (e.g., episodes of child's distress and illness are minimized or ridiculed) as an active turning away of the child's expression of need and attachment. The caregiver seems to prefer the child's absence by acting on feelings of anger, enmity, irritation, bitterness, or resentment. The child may feel disliked, avoided, and unwanted. Items referring to *Role reversal* behaviors label caregiver who seeks comfort, intimacy, and physical or psychological unmet needs from the child. Child's presence and attention seem essential for the caregiver's sense of welfare. The child is thus expected to take the role of a parent, spouse, or peer toward the caregiver and attempts to reduce his/her distress. Items of *Psychological abuse* assess verbal and demonstrative acts by the caregiver intended to cause psychological pain or fear, as excessive and repeated ridiculing, blaming, insults, isolation, threats, and coercive attitudes toward the child. The caregiver's intent seems the control or intimidation of the child, and items range from shaming or embarrassing the child to parental alienation syndrome, intrusive and excessive medical care, or emotional cruelties (e.g., threat to hurt the child or the child's loved one or to force him/her to violate the law). Questions regarding *Physical abuse* inquire about the occurrence of physically aggressive behaviors (e.g., hitting, grabbing, knocking down, beating with an object, tying, and burning), resulting injuries (e.g., cuts, bruises, broken bones, and burns), and medical treatment needed. Supplementary queries probe regarding the nonabusive caregiver's responses to maltreatment perpetrated by other household members (e.g., defending, reassuring, and comforting the child). The *Sexual abuse* domain includes any sexual intercourse that involves (e.g., inappropriate touching, kissing, fondling, and penetration) or does not involve (e.g., voyeurism, exposure to erotic language, and participation in pornographic activities) physical contact. In addition, items explore if the participant has been threatened not to recount, if the caregiver did not believe the report, or failed to comfort and protect the child from abuse. Items regarding the *Witnessing domestic violence* area describe verbal/emotional abuse and aggressive behaviors that occur between parents to which the child is exposed. Finally, significant *Separations* and *Losses* up to age 14 are explored.

### Participants

The study has been conducted among 229 individuals, including participants at high-risk and with psychiatric diagnoses (dissociative disorders, *N* = 14; parents of maltreated children, *N* = 53; gender identity disorder in the procedure of sex reassignment surgery, *N* = 41; personality disorders, *N* = 42), and a nonclinical sample (*N* = 79). Subjects were recruited from clinical centers in Rome (Italy) and took part in different research projects (Mirizio et al., 2011; Farina et al., 2014; Speranza and Maggiora Vergano, 2015; Lingiardi et al., submitted). Although important differences characterize participants within the clinical/at-risk sample, we decided to combine those groups due to their history of trauma exposure as suggested by literature. Indeed, several studies highlighted early trauma in dissociative patients (Dutra et al., 2009; Schmahl et al., 2010), parents of maltreated children (Dixon et al., 2005a,b, 2009), participants with gender identity disorder (Kersting et al., 2003; Gehring and Knudson, 2005; Veale et al., 2010), and those with personality disorders (Afifi et al., 2011). The combined clinical/at-risk sample (*N* = 150) was composed of 37 men, 72 women, 27 male-to-female and 15 female-to-male transsexuals, ranging from 16 to 62 years of age (*M* = 30.44; *SD* = 11.24), and mostly Italians (94%). The majority of nonclinical participants were females (82%) and Italian (99%) with the mean age of 31.87 years (*SD* = 5.76) and age range of 25–65 years. The nonclinical participants were recruited from previous longitudinal studies regarding the quality of parenting (Ammaniti et al., 2005, 2006), representing the control group which had no depressive or psychosocial risks.

The overall sample was recruited from different research studies expressively asking for a participation secured through a written informed consent procedure that required active consent from participants. The current study has been attended within the Ph.D. context at the Department of Dynamic and Clinical Psychology (Faculty of Medicine and Psychology, Sapienza University of Rome) and received the approval from the Ethical Committee of the Department.

### Instruments

#### Adult attachment interview

Adult Attachment Interview (AAI; George et al., 1984; Main et al., 2003). The AAI is a semi-structured interview, an hour-long protocol, which explores adult's mental representations of attachment while discussing childhood experiences. After an overview of the family composition, respondents are asked to describe their early relationship with each parent, supplying five adjectives to be supported by providing specific descriptive incidents. Interviewees are additionally probed regarding situations of distress (e.g., emotionally upset, hurt, or ill), and instances of threat or abuse. Following questions concern reaction to separations from the caregiver and significant losses. Two types of variables are rated on 9-point Likert scales. Narratives of parent–child relationships are coded on scales for “probable childhood experiences” which describe rejecting, involving/role reversing, neglecting when present, pressuring to achieve, and loving behaviors of each attachment figure. Likewise, “present state of mind” with respect to attachment is rated on scales defining idealization of parents, dismissing derogation of attachment figures or relationships, insistence on lack of recall, fear of loss of the child, passivity or vagueness in discourse, current involving/preoccupying anger toward caregivers, metacognitive monitoring, coherence of transcript, and overall coherence of mind. AAI scoring system is based on the participant's ability to produce coherent narratives regarding childhood experiences with caregivers, thus classifying interviewee as Secure/Autonomous (F), Dismissing (Ds), Preoccupied with respect to attachment (E), or “Cannot classify” category (CC) when a global breakdown in the organization of discourse arises. An interview may also be assigned an Unresolved/disorganized state of mind (Ud) concerning past abuse or loss in association with a best-fitting primary classification. Psychometric testing and meta-analyses have provided evidence of stability and discriminant and predictive validity of the AAI in both clinical and nonclinical populations (Bakermans-Kranenburg and van IJzendoorn, 1993, 2009; Sagi et al., 1994; van IJzendoorn, 1995; Roisman et al., 2007; Hesse, 2008; van IJzendoorn and Bakermans-Kranenburg, 2008). Each Adult Attachment Interview was conducted by researchers trained by A.M. Speranza to administer the AAI, recorded and transcribed verbatim. The interviews were classified using Main et al. (2003) coding system^2^ by the first and the third author who are certified AAI coders. A hundred and thirty-five transcripts (59%) were double-coded, blind to subject conditions: percent agreement for the 4-way classification (F, Ds, E, Ud/CC) was 81% (*k* = 0.77). When there was disagreement, a third independent coder also categorized the transcript, with final agreement reached after discussion by all three coders.

#### Complex Trauma Questionnaire

Complex Trauma Questionnaire (ComplexTQ). The ComplexTQ is a 70-item scale for the retrospective assessment of multi-type maltreatment, measuring lack of care (physical and emotional neglect), abuse (psychological, physical, and sexual abuse), and other traumatic experiences, such as rejection, role reversal, exposure to domestic violence, separations, and losses. The instrument is available in two different versions, clinician and self-report. The questionnaire assesses adverse experiences from childhood to usage of 14 years separately involving maternal, paternal, and other attachment figures. The clinician version, compiled by the first and the third author, requires approximately 15–20 min to complete (depending on the length of the interview's transcript) and scores for presence and frequency of traumatic experiences in each domain are automatically provided by the software.

### Statistical analyses

#### Inter-rater reliability

Although the data used in the present study were collected by a single rater who evaluated all transcripts, we conducted a preliminary analysis aimed to test rater's ability to code data. This analysis was based on a pilot study of 54 participants later included in this study, each evaluated by the first and the third author of the manuscript, blind to each other, using the complete 70-item set. The inter-rater reliability for ComplexTQ ratings was assessed based on the Intraclass Correlation Coefficient (ICC), a measure of similarity of ComplexTQ ratings made by independent coders. The analysis yielded good inter-rater reliability for the 70 items (ICC-s = 0.85 and 0.89 for items concerning mother and father, respectively) as well as for the items retained after factor analysis (ICC-s = 0.81 and 0.87 for items concerning mother and father, respectively).

#### Factor analysis

##### Polychoric correlations

A preliminary descriptive analyses of ComplexTQ items revealed that 27 and 30 items, for patients' traumatic experience with mother and father, respectively, had near zero variance in the samples studied. These items were excluded from the analyses that were completed on 40 and 37 items. However, the retained items not only were ordered categorical variables but the inspection of the item response pattern also revealed in most cases an asymmetric response distribution. Standard factor analysis assumes a multivariate normal distribution and item distributions that approach an equal intervals scale. As both the assumptions were violated, we analyzed the polychoric correlation matrix, instead of the standard Pearson's correlation matrix. This procedure is strongly recommended for ordered categorical items from two to five response categories (Panter et al., 1997; Holgado-Tello et al., 2008; Timmerman and Lorenzo-Seva, 2011; Garrido et al., 2013). Specifically, a polychoric correlation is based on the assumption that each of the ordered categorical items represents an approximation of an underlying continuous variable. Accordingly, the computational procedure estimates what would be the correlation between these underlying variables based on the collected empirical approximation. An unweighted least square factor analysis with promax rotation was conducted by Factor 9.3 (Lorenzo-Seva and Ferrando, 2006). The Kaiser–Meyer–Olkin measure of sampling adequacy attained a fairly high value for both mother and father datasets (KMO = 0.72 and 0.80, respectively), thus showing that the sample correlation matrix was appropriate for factor analysis to proceed.

##### Factor retention criteria

Despite there being no “gold standard” for determining the “true” number of factors to retain in exploratory factor analysis, we integrated the scree-plot with information from Minimum Average Partial (MAP) and Parallel Analysis (PA), which are considered the most accurate diagnostics (Ruscio and Roche, 2012). The MAP test is based on a series of factor analyses, each followed up by a quantitative assessment of partial correlations. Specifically, as the first step, a factor analysis is performed and the first factor is partialed out of the correlation matrix among questionnaire items. Then, the squared off-diagonals partial correlations are averaged to obtain a summary index of the variance in the correlation matrix that is due to systematic sources. As the second step, both first and second extracted factors are partialed out and again the squared off-diagonals partial correlations are averaged, and this procedure is repeated up to k-1 factors are partialed out. The MAP test lines up the average squared partial correlation indexes obtained on each step, and the minimum value is considered as indication of the appropriate number of factors in the dataset. PA is based on comparing eigenvalues resulting from a factor analysis of real data to those resulting from a Monte Carlo simulation study. Specifically, repeated factor analyses are performed on a large number of randomly generated data matrices (e.g., 1000) that are parallel to the real data (i.e., have the same number of variables and cases). Factors whose eigenvalues are greater than the average random ones are deemed as reflecting systematic sources and therefore, retained as meaningful psychological dimensions (for a recent review of factor retention criteria see Courtney, 2013).

#### ROC curve analysis

The clinical validity of the ComplexTQ was assessed by plotting the performance of each factor's derived scale score as a classifier of clinical vs. nonclinical group membership (see participants). A Receiver Operating Characteristic (ROC) curve analysis of these plots was performed by SPSS 23 to determine the optimal threshold for ComplexTQ. On each analysis, the “true positive” rate at different threshold is plotted against the “false positive” rate and the resulting curve connecting data points was drawn. The area under the curve (AUC) is a measure of the scale score classification accuracy, and its statistical significance supports clinical validity.

#### Convergent validity

The transcripts were also coded through AAI scales for inferred experiences in infancy with mother and father. Convergent validity of ComplexTQ scales was then assessed as correlation coefficients with AAI scales.

## Results

### Factor analysis

The analysis of ComplexTQ items yielded 16 and 15 eigenvalues that were greater than one for mother and father datasets, respectively. Regarding mother dataset, the scree-plot showed three major drops in eigenvalue size after the third, the sixth, and the eighth eigenvalue. The MAP test suggested two factors to be retained, while the PA criterion indicated six factors. Regarding father dataset, the scree-plot showed two major drops in eigenvalue size after the second and the fifth eigenvalue. The MAP test suggested three factors to be retained, while the PA criterion indicated five factors. Thus, there is agreement between scree-plot and PA on six and five factors for mother and father, respectively. Besides that, the six- and five-factor solutions also had a better fit than solutions based on a smaller number of factors (GFI-s = 0.94 and 0.95 for mother and father datasets, respectively).

The mother dataset showed the following factors accounting for 49% of the variance in trauma experience (see Table 1). (F1) Role Reversal loaded on eight items evaluating the extent to which the mother placed herself in the center of child's attention, involving the child in her physical and/or psychological care, or making the child feel responsible for her own wellbeing. Moreover, other items inform about situations of domestic conflict and violence witnessed by the child. (F2) Physical Abuse loaded on seven items describing forms of maltreatment and physical abuse perpetrated by the mother. Furthermore, the caregiver tended to deride or get angry at the child's signals of attachment needs, arousing child's fear and worries. (F3) Psychological Abuse/Rejection loaded on eight items assessing rejection and avoidance of the child, especially child's expression of affection, attention, dependence, and attachment. The mother's abuse and aversion, exhibited through criticism, insults, or humiliations, made the child feel unwanted and disliked. (F4) Emotional Neglect loaded on seven items describing indifference, inattention, or psychological unavailability to the child's emotional needs. It includes failure to provide nurturance or companionship within child–caregiver relationship and lack of emotional support to the child when expecting comfort and reassurance. Therefore, the child may not rely upon the mother for sharing difficult or painful experiences. (F5) Failure of Protection loaded on three items regarding a parent who abdicated the role of caregiving, by failing to defend and reassure the child in abusive situations perpetrated by other caretaking figures. (F6) Material Neglect loaded on seven items considering physical neglect (e.g., failure to provide adequate food, clothing, shelter, and medical care), educational neglect (e.g., failure to ensure proper education and learning opportunities), and supervisory/social neglect (e.g., unawareness/inattention to child's whereabouts and socializing).

**Table 1:** **Six-factor model of ComplexTQ items for the mother**.

**Items**		**F1**	**F2**	**F3**	**F4**	**F5**	**F6**
24	The mother depended upon the child	**0.96**					
21	The mother involved the child in her own physical or psychological needs	**0.67**					
64	The child was exposed to domestic violence	**0.51**					
22	The mother sought the child's proximity and attention for her own wellbeing	**0.48**					
63	The child was exposed to family physical fights with throwing objects or blows	**0.42**					
27	The child felt responsible for the mother or desired to protect her	**0.41**					
23	The mother delegated her assignments and duties to the child	**0.32**					
25	The mother induced feelings of guilt and preoccupation to the child	0.11					
48	The mother left marks when striking the child		**0.91**				
47	The mother hit with or threw objects at the child		**0.83**				
46	The mother beat, pushed or grabbed the child with strength		**0.50**				
29	The child was scared when the mother screamed for reprimanding		**0.43**				
19	The mother mocked or got angry with the child when he/she was scared, injured, ill, or emotionally distressed		**0.27**				
15	The mother showed favoritism toward the child's siblings		0.04				
18	The mother minimized episodes of child's distress, fear, injury, or illness		0.03				
16	The mother often and/or unfairly criticized the child			**0.73**			
30	The mother insulted or swore at the child			**0.73**			
31	The mother denigrated and humiliated the child			**0.68**			
17	The mother showed indifference, annoyance, and active dislike toward the child			**0.54**			
14	The child thought that the mother was extremely difficult to please			**0.29**			
20	The child felt him/herself unwanted or refused by the mother			**0.23**			
28	The mother prohibited to the child to be autonomous and feel free to behave			0.17			
41	The mother threatened to abandon the child			0.04			
3	The mother did not show physical/verbal expressions of affection toward the child				**0.71**		
7	The mother was not concerned about the child's problems				**0.66**		
8	The mother did not provide support and reassurance when the child was scared or distressed				**0.64**		
12	The child did not feel comfortable to confide in the mother about difficult/painful experiences				**0.57**		
6	The mother did not support the child in his/her activities and was not proud of him/her				**0.52**		
13	The child felt neglected or ignored by the mother due her being deep into own activities				**0.43**		
5	The mother was not glad to spend time with the child or have him/her around				**0.42**		
52	The mother did not defend the child when he/she was physically abused					**0.92**	
53	The mother did not give support when the child was physically abused					**0.88**	
62	The child was exposed to family discussions and arguing with screams and insults					**0.23**	
11	The mother was not interested in how the child used to spend his/her spare time						**0.87**
1	The mother was not concerned about the child's basic physical needs						**0.62**
2	The mother did not care that the child receive a proper education						**0.53**
4	The mother was not concerned about the child's socializing						**0.52**
9	The mother was not concerned and soothing or tried to make the child feel better when he/she was injured						**0.31**
69	The child was placed in foster care or institution						0.19
10	The mother was not concerned and soothing or tried to make the child feel better when he/she was ill						0.17
	Cronbach's α		0.74	0.72	0.73	0.85	0.65
	Composite Reliability (Mislevy and Bock, 1990)		0.93	0.92	0.81	0.80	0.90
	Explained variance (%)		12	10	9	6	6

*Items retained for ComplexTQ factor-derived scales in bold*.

The father dataset showed similar factors that emerged, accounting for 51% of the variance in trauma experience (see Table 2). Unlike the mother dataset, no role-reversal factor was identified, the factor ordering was different and the factors loaded on different number of items but preserved the original meaning: (F1) Emotional Neglect (9 items), (F2) Failure of Protection (3 items), (F3) Material Neglect (7 items), (F4) Psychological Abuse/Rejection (7 items), and (F5) Physical Abuse (11 items).

**Table 2:** **Five-factor model of ComplexTQ items for the father**.

**Items**		**F1**	**F2**	**F3**	**F4**	**F5**
7	The father was not concerned about the child's problems	**0.82**				
12	The child did not feel comfortable to confide in the father about difficult/painful experiences	**0.74**				
8	The father did not provide support and reassurance when the child was scared or distressed	**0.61**				
17	The father showed indifference, annoyance, and active dislike toward the child	**0.49**				
3	The father did not show physical/verbal expressions of affection toward the child	**0.47**				
6	The father did not support the child in his/her activities and was not proud of him/her	**0.41**				
13	The child felt neglected or ignored by the father due his being deep into own activities	**0.38**				
5	The father was not glad to spend time with the child or have him/her around	**0.37**				
15	The father showed favoritism toward the child's siblings	0.06				
52	The father did not defend the child when he/she was physically abused		**0.99**			
53	The father did not give support when the child was physically abused		**0.93**			
14	The child thought that the father was extremely difficult to please		0.13			
11	The father was not interested in how the child used to spend his/her spare time			**0.81**		
4	The father was not concerned about the child's socializing			**0.67**		
9	The father was not concerned and soothing or tried to make the child feel better when he/she was injured			**0.56**		
10	The father was not concerned and soothing or tried to make the child feel better when he/she was ill			**0.54**		
1	The father was not concerned about the child's basic physical needs			**0.50**		
68	The child was abandoned from the father			**0.49**		
2	The father did not care that the child receive a proper education			**0.44**		
30	The father insulted or swore at the child				**0.96**	
16	The father often and/or unfairly criticized the child				**0.81**	
31	The father denigrated and humiliated the child				**0.69**	
29	The child was scared when the father screamed for reprimanding				**0.25**	
20	The child felt him/herself unwanted or refused by the father				**0.25**	
28	The father prohibited to the child to be autonomous and feel free to behave				0.11	
19	The father mocked or got angry with the child when he/she was scared, injured, ill, or emotionally distressed				0.01	
48	The father left marks when striking the child					**0.80**
47	The father hit with or threw objects at the child					**0.67**
51	The child thought that received punishments were cruel					**0.65**
64	The child was exposed to domestic violence					**0.63**
46	The father beat, pushed or grabbed the child with strength					**0.57**
63	The child was exposed to family physical fights with throwing objects or blows					**0.53**
62	The child was exposed to family discussions and arguing with screams and insults					**0.22**
18	The father minimized episodes of child's distress, fear, injury, or illness					0.06
27	The child felt responsible for the father or desired to protect him					0.05
41	The father threatened to abandon the child					0.02
69	The child was placed in foster care or institution					0.01
	Cronbach's α	0.87	0.99	0.85	0.75	0.78
	Composite Reliability (Mislevy and Bock, 1990)	0.84	0.99	0.85	0.91	0.85
	Explained variance (%)	16	13	8	7	7

*Items retained for ComplexTQ factor-derived scales in bold*.

### ComplexTQ factor-derived scales

Factor markers with factor loadings greater than 0.20 were selected based on inspection of the factor loading matrix. Our aim was to retain most representative items for each factor and to discard items that failed to load any factors. Then, selected items were resubmitted to factor analysis to verify whether item selection biased factor interpretation. Since no substantial changes in factor labeling and content emerged, ComplexTQ factor-derived scales were based on 33 and 29 items respectively for mother and father datasets (reported in bold font on Tables 1, 2).

### ROC curves

As one can see from Table 3, ComplexTQ factor-derived scales were used as predictors of group membership and discriminant properties were assessed as AUC-s. For mother and father data, ComplexTQ scores were significantly different for clinical and nonclinical participants. In particular, fairly high AUC values were found for the Emotional Neglect scale for both the mother and the father, while Material Neglect attained the fair AUC threshold for father only. It is worth noting that AUC values for the total scores approached the standard for good diagnostic accuracy.

**Table 3:** **Analysis of ROC curves to assess diagnostic accuracy for ComplexTQ factor-derived scales and total scores**.

	**Factors**	**Sensitivity**	**Specificity**	**Positive if >**	**AUC**	**Standard Error**	***p***
Mother	F1: Role reversal	0.530	0.785	1	0.673	0.036	<0.001
	F2: Physical abuse	0.503	0.658	1	0.611	0.037	<0.01
	F3: Psychological abuse/reject	0.644	0.684	1	0.691	0.035	<0.001
	F4: Emotional neglect	0.651	0.709	5	0.722	0.035	<0.001
	F5: Failure of protection	0.456	0.823	1	0.647	0.037	<0.001
	F6: Material neglect	0.450	0.747	1	0.664	0.038	<0.001
	Total	0.711	0.759	9	0.789	0.031	<0.001
Father	F1: Emotional neglect	0.651	0.734	5	0.767	0.031	<0.001
	F2: Failure of protection	–	–	–	0.577	0.038	n.s.
	F3: Material neglect	0.624	0.722	2	0.727	0.034	<0.001
	F4: Psychological abuse/reject	0.510	0.747	1	0.647	0.037	<0.001
	F5: Physical abuse	0.550	0.709	1	0.662	0.036	<0.001
	Total	0.678	0.797	9	0.789	0.031	<0.001

### Convergent validity

As shown in Table 4, the mother's ComplexTQ factors were correlated with AAI scales for inferred experiences. AAI “Pressure to achieve” probable experience scale was omitted as no correlations were found with ComplexTQ factor-derived scales. We reviewed correlation coefficients following Cohen's (1988) “rule of thumb.” There was a very large overlapping between ComplexTQ Role Reversal factor and AAI Involving/role reversing scale. Physical Abuse factor showed a medium-large correlation with AAI Rejecting scale. Psychological Abuse factor showed a large and small-medium correlation with AAI Rejecting and Neglecting scales, respectively. Emotional Neglect factor showed a very large correlation with AAI Neglecting and Rejecting scales. Failure of Protection factor showed a small-medium correlation with AAI Rejecting and Neglecting scales. Material Neglect showed a large correlation with AAI Rejecting and Neglecting scales. It is noteworthy that all ComplexTQ factors were negatively associated with the AAI Loving scale, although the effect size varied from small (Role Reversal) to very large (Emotional Neglect).

**Table 4:** **Correlations with AAI scales for inferred experiences with mother**.

**Mother**	**Loving**	**Rejecting**	**Neglecting**	**Involving/role-reversing**
F1: Role reversal	−0.19^*^	0.05	0.10	0.74^***^
F2: Physical abuse	−0.31^***^	0.38^***^	0.06	0.02
F3: Psychological abuse/reject	−0.41^***^	0.53^***^	0.27^**^	−0.01
F4: Emotional neglect	−0.76^***^	0.72^***^	0.74^***^	0.14
F5: Failure of protection	−0.29^**^	0.26^**^	0.25^**^	0.08
F6: Material neglect	−0.58^***^	0.59^***^	0.55^***^	0.08

* *p < 0.05*;

** *p < 0.01*;

*** *p < 0.001*.

The father's ComplexTQ factors (see Table 5) had similar correlation patterns as mother for Neglect and Psychological Abuse factors. Instead, Failure of Protection factor was significant only with AAI Rejecting scale showing a small effect size, and Physical Abuse factor was medium-large with both AAI Neglecting and Rejecting scales. All ComplexTQ factors were negatively associated with the AAI Loving scale.

**Table 5:** **Correlations with AAI scales for inferred experiences with father**.

**Father**	**Loving**	**Rejecting**	**Neglecting**	**Involving/role-reversing**
F1: Emotional neglect	−0.78^***^	0.79^***^	0.78^***^	0.05
F2: Failure of protection	−0.19^*^	0.25^*^	0.11	0.03
F3: Material neglect	−0.61^***^	0.64^***^	0.63^***^	0.01
F4: Psychological abuse/reject	−0.42^***^	0.54^***^	0.27^**^	−0.04
F5: Physical abuse	−0.44^***^	0.47^***^	0.31^**^	0.12

* *p < 0.05*;

** *p < 0.01*;

*** *p < 0.001*.

## Discussion

In response to the need for psychometrically based trauma instruments, this study describes the development and the preliminary psychometric properties of the ComplexTQ, confirming satisfactory reliability and validity of the measure.

This study mainly aimed to evaluate the validity of the ComplexTQ. Multiple trauma dimensions were validated through factor analysis, which provided coherent outcomes for both mother and father ratings. Dealing with a priori developed ComplexTQ scales, Neglect subscale was divided into a material form—reflecting physical, educational, and supervisory/social negligence—and the caregiver's unavailability or inattention which mainly involves emotional level. In this regard, several studies highlighted the need for an articulated conceptualization of neglect due to the heterogeneity of the phenomenon (e.g., Dubowitz et al., 1993; Slack et al., 2003), since they observed different consequences on child's well-being and different approaches to primary prevention and treatment with respect to the form in which neglect is declined (Straus and Kaufman Kantor, 2005). Despite the need of a comprehensive definition and assessment, the choice of splitting neglect in different dimensions has been noticed in few childhood trauma measures, as the Childhood Trauma Questionnaire (CTQ; Bernstein et al., 1994), the Interview for Traumatic Events in Childhood (ITEC; Lobbestael et al., 2009), the questionnaire used in the Adverse Childhood Experiences Study (Felitti et al., 1998), or the Multidimensional Neglectful Behavior Scale (MNBS; Straus et al., 1995). Conversely, Rejection and Psychological Abuse aspects converged into a single factor, therefore associating caregiver's cold and hostile attitude to manifestations of abuse which cause psychological suffering and fear with the intent to control and intimidate the child. This process of inclusion also arose between Role Reversal and Witnessing Domestic Violence items concerning early experiences with the mother. In this matter, it is interesting to underline that the child's exposure to conflicts and violent episodes that occurred within the family were used to represent a form of psychological abuse. Only recently, it has been recognized as a type of maltreatment (Higgins, 2004). Due to the emerged outcomes of this study, we can speculate that deep in an intra-familiar violent climate—in which the father often constitutes the abusing figure—the child may feel responsible for and involved in the physical or psychological care of the caregiver who is subjected to aggressions. Effects of exposure to domestic violence, reflected on the child's desire to comfort and protect the mother, seems to endure even when episodes of abuse end (Macfie et al., 2008). In a similar vein, it has not been pinpointed a factor including aspects of Role Reversal in relation to childhood experience with the father. Moreover, referring to early relationship with both the caregivers, factor analysis did not identify a Sexual Abuse factor. This result is presumably due to the lack of frequency of sexual abuse episodes in the analyzed reports. Based on our data, we may not exclude that sexual abuse is important to assess, but rather it is underrepresented as an early traumatic experience in participants of this study. Finally, a substantial development of our investigation consisted of an emergent new factor, defined as Failure of Protection of the child, which mainly arises when physical abuse perpetrated by another caregiver occurs. This factor identification is in accordance with the context described by George and Solomon (1996); George and Solomon (2008), referring to a mother who psychologically abdicates her caregiving role whenever the child shows attachment needs. Therefore, it is reasonable to believe that many of the psychological maltreatment correlates are not an exclusive effect of the traumatic experiences occurrence, but rather are a consequence of the impact of caregiver's inadequate dealing with his/her relationship with the child and affect regulation needs.

The ComplexTQ's multifactorial structure has the advantage to cover a wide range of traumatic experiences occurred in early attachment relationships, including aspects of neglect that have been for a long time poorly considered by child abuse research—“*The Neglect of Neglect*” (Wolock and Horowitz, 1984; Dubowitz, 1994, 2007; Kaplan et al., 1999; Stoltenborgh et al., 2013). Therefore, ComplexTQ enables identifying and assessing multifaceted aspects of trauma in early life, thereby grasping a dysfunctional and disorganized emotional climate rather than focusing on single or non-relational adverse experiences and on a specific dimension of trauma. An accurate identification of multiple traumatic forms appears to be essential, even at a prevention level, considering the consequences in terms of intergenerational transmission of trauma and developmental trajectories, in which scientific literature underlines the transversal nature of trauma as a risk factor for later psychopathology. Research on therapeutic treatment of maltreated children has not yet highlighted a distinction for complex trauma victims, which would allow establishing a different response to focused intervention for the co-occurrence of cumulative traumatic experiences (Harvey and Taylor, 2010; Ford et al., 2012). Studies comprising adult population suggest a adjustment oriented therapeutic process using the results of early exposure to multiple traumas (Ford and Kidd, 1998; Nemeroff et al., 2003).

Concerning the diagnostic accuracy, ComplexTQ total scores discriminated clinical/at-risk vs. nonclinical participants, thus being a valid measure to evaluate traumatic experiences occurred in early attachment relationships. This aspect seems to be particularly relevant, since different studies emphasized the utility of some instruments (e.g., CTQ and ACE Questionnaire) in evaluating high levels of early trauma exposure and in associating them to a major risk of developing a later psychopathology (Teicher and Parigger, 2015).

Regarding correlation with AAI scales for inferred experiences, except Role Reversal and Failure to Protection, factor-derived ComplexTQ scales deal with same aspects of rejecting and neglecting, providing a more articulated vision and, therefore, converging with the latest scientific studies which recognize multifaceted expressions of constructs (e.g., Hart et al., 2002; Dubowitz et al., 2005; Baker, 2009). Moreover, all factors were negatively associated with the AAI Loving scale, which describes an active process of caregiver's involvement, support, and nurturance with respect to the child. Similar to active forms of maltreatment, lack of emotional support from caregivers has been associated with externalizing and internalizing symptoms across life span (McCarty et al., 2004; Shaw et al., 2004).

## Limitations and final remarks

The limitations of the study displayed future directions for the confirmatory validation and use of the ComplexTQ. The present study outcomes are based on a factor analysis that needs additional confirmation with an independent and wider sample. A larger sample is also required to unable an invariance analysis by gender in the relationship with both caregivers. Since this is a preliminary study, all 70 items in the current version should be retained, and choice to exclude the less representative items from scales should be delayed till future analyses. A second limitation concerns the absence of convergence with an independent retrospective measure of childhood trauma, which could therefore better verify ComplexTQ's construct validity. The intraclass correlation assessed on 54 questionnaires may represent another limitation, although it is not unusual that the inter-rater reliability is tested on a subset of participants to be generalized to the full sample (see Hallgren, 2012). Moreover, among the different parameters used to quantify the traumas, ComplexTQ does not inquire about other aspects, such as age at the onset and the duration of traumatic experiences, which seem to have an impact on later psychopathology (Bifulco et al., 2002; Roy and Perry, 2004; English et al., 2005). Finally, diagnostic accuracy has been verified comparing clinical/at-risk and nonclinical participants, therefore not disclosing if ComplexTQ factors could be useful to discriminate different types of psychopathology. To present the state of affairs, we can consider ComplexTQ scores only as trans-diagnostic indicators, hence related to several aspects of psychopathology.

The ComplexTQ clinician report version has been applied to AAI transcripts, a protocol which represents an emotional stimulus able to activate attachment system and conceived by the authors as an attempt to “surprise the unconscious” (Main, 1991, p. 141). Considering this premise, ComplexTQ offers the facilitation, compared with the AAI-based studies, to use the interview as an instrument that gives specific and detailed information about the presence and severity of trauma in participant history, without requiring training for the reliability to code attachment mental states. Moreover, ComplexTQ enables to distinguish with which attachment figure the participant experienced specific traumatic events. This specificity allows evaluating the influence of other significant figures and, moreover, the different effects of trauma on development due to the relevance of the caregiver's gender regarding later outcomes (Schore, 1999; Briere and Rickards, 2007). In addition to the nature of trauma and the perpetrator's identity, the scoring system also enables to assess the occurrence as well as the frequency of each different type of maltreatment. Finally, the questionnaire is easy to complete and to code, owing to a program specifically created for this research that shows a profile of the child relational history. Unlike most of the studies assessing early interpersonal trauma (Briere et al., 2012), reported results were based on heterogeneous clinical groups and nonclinical sample, permitting more generalizability of findings. Therefore, ComplexTQ is a promising tool for the retrospective measurement of early trauma which can be used for clinical and research purposes and enables to discriminate clinical and nonclinical outcomes, supplying a contribution to the understanding of the link between childhood trauma and psychopathology.

## Author contributions

We state that all authors have participated in the work with a substantial contribution to conception, design, acquisition, analysis and interpretation of data. Moreover, all authors have been involved in drafting the article and approved the final version of the manuscript. Agreement has been accountable for all aspects of the manuscript in ensuring that questions related to the accuracy or integrity of any part of the work are appropriately investigated and resolved.

### Conflict of interest statement

The authors declare that the research was conducted in the absence of any commercial or financial relationships that could be construed as a potential conflict of interest.

## Footnotes

^1^The self-report version of ComplexTQ is not presented in this paper.

^2^C. Maggiora Vergano has been trained to reliability at the AAI Training Institute of Rome, 2010, by D. Jacobvitz and N. Dazzi; A. M. Speranza at the AAI Training Institute of Rome, 1990, by M. Main and E. Hesse; C. Trentini (third coder) at the AAI Training Institute of Rome, 2008, by D. Jacobvitz and N. Dazzi.
